# Antitumor Activity of Alloy and Core-Shell-Type Bimetallic AgAu Nanoparticles

**DOI:** 10.1186/s11671-017-2112-y

**Published:** 2017-05-04

**Authors:** Igor Shmarakov, Iuliia Mukha, Nadiia Vityuk, Vira Borschovetska, Nelya Zhyshchynska, Galyna Grodzyuk, Anna Eremenko

**Affiliations:** 1 Department of Biochemistry and Biotechnology, Yuriy Fedkovych Chernivtsi National University, Kotsuybynskyiy St., 2, Chernivtsi, 58012 Ukraine; 2 Laboratory of Photonics of Nanosized Oxide Systems, Chuiko Institute of Surface Chemistry, National Academy of Sciences of Ukraine, General Naumov St., 17, Kyiv, 03164 Ukraine; 3 L.V. Pisarzhevskii Institute of the Physical Chemistry, National Academy of Sciences of Ukraine, Kyiv, Ukraine; 4 NanoMedTech LLC, Kyiv, Ukraine

**Keywords:** Gold, Silver, Nanoparticles, Anticancer activity, Oxidative stress, 78.67.Bf (Nanocrystals, nanoparticles, and nanoclusters), 87.85.Rs (Nanotechnologies-applications), 87.85.em (Tissue damage)

## Abstract

Nanoparticles (NPs) of noble metals, namely gold and silver, remain promising anticancer agents capable of enhancing current surgery- and chemotherapeutic-based approaches in cancer treatment. Bimetallic AgAu composition can be used as a more effective agent due to the synergetic effect. Among the physicochemical parameters affecting gold and silver nanoparticle biological activity, a primary concern relates to their size, shape, composition, charge, etc. However, the impact of metal components/composition as well as metal topological distribution within NPs is incompletely characterized and remains to be further elucidated and clarified. In the present work, we tested a series of colloidal solutions of AgAu NPs of alloy and core-shell type for an antitumor activity depending on metal molar ratios (Ag:Au = 1:1; 1:3; 3:1) and topological distribution of gold and silver within NPs (Au_core_Ag_shell_; Ag_core_Au_shell_). The efficacy at which an administration of the gold and silver NPs inhibits mouse Lewis lung carcinoma (LLC) growth in vivo was compared. The data suggest that in vivo antitumor activity of the studied NPs strongly depends on gold and silver interaction arising from their ordered topological distribution. NPs with Ag core covered by Au shell were the most effective among the NPs tested towards LLC tumor growth and metastasizing inhibition. Our data show that among the NPs tested in this study, Ag_core_Au_shell_ NPs may serve as a suitable anticancerous prototype.

## Background

Rapid advances in nanomedicine have generated an increasing number of potential diagnostic and therapeutic applications of nanoparticles in recent years [1, 2]. Among the different types of nanoparticles, metal nanoparticles including gold and silver have widespread use across the biomedical sciences [3, 4]. Nanoparticles (NPs) based on noble metals remain promising anticancer agents capable of enhancing existing surgery and chemotherapeutic-based approaches in cancer treatment. These NPs are being extensively used in a diagnostics and anticancer therapy due to intrinsic noble metal properties including ultramicroscopic size, large surface area-to-volume ratio, and consequently their electronic and optical properties as well as high reactivity and stability [4]. In vitro and in vivo studies demonstrated that gold and silver NPs have strong antitumor activities [3, 5–7]. Bimetallic AgAu NPs are advantageous because they combine and resemble the unique properties of both gold and silver in one nanoparticle that are distinctly different from those inherent to individual components [1, 8]. Such physicochemical parameters as size, shape, composition, and charge greatly influence biological activity of NPs [9–12]. These properties of bimetallic AgAu NPs have been also shown to be advantageous in a development of aptamer-based biosensors [13]. However, many aspects of gold and silver NP-based systems remain unexplored, limiting the efficiency of their application. For instance, the impact of metal composition as well as metal topological distribution within NPs is incompletely characterized and remains to be further elucidated and clarified. An inclusion of different metallic compositions has been shown to significantly affect reactive oxygen species (ROS) formation and has been suggested to be a major cause of different cellular disruptions and cell death enhancement observed in cells exposed to nanoparticles [12]. Our previous study demonstrated that combination of both gold and silver in one bimetallic NP confers advantageous biological activities and low toxicity in vivo [14]. In the present work, we test the ability of various bimetallic AgAu NPs to inhibit the metastatic tumor growth in vivo, employing mouse Lewis lung carcinoma (LLC) as a model. Aiming at expanding our knowledge on the AgAu nanoparticles biological activity, NPs were tested for an antitumor activity depending on metal molar ratios (Ag:Au = 1:1; 1:3; 3:1) and topological distribution of gold and silver within NPs (Au_core_Ag_shell_; Ag_core_Au_shell_). The data, revealed in the present study, emphasize that both parameters, metal molar ratio as well as metal topological distribution within bimetallic gold and silver nanoparticles, are critical features of NPs and can affect their antitumor activity. Among the NPs tested in this study, Ag_core_Au_shell_ NPs may serve as the most suitable prototype for the development of an anticarcinomatous agent.

## Methods

### Nanoparticle Synthesis and Characterization

Colloidal solutions of bimetallic silver and gold NPs were obtained via chemical reduction of silver nitrate and tetrachlorauratic acid (AgNO_3_ and HAuCl_4_, Merck, Germany) with amino acid tryptophan (Trp, SC12-20120713, China). The components interacted in a molar ratio Trp:M = 2:1. The concentration of metal in the resulting solution was C(M) = 10^-4^M followed by concentrating it to 10^−3^ M. Bimetallic NPs of alloy type were obtained via simultaneous reduction of metal ions using initial metal molar ratios of Ag:Au = 3:1, 1:1, and 1:3, indicated as AgAu(3:1), AgAu(1:1), and AgAu(1:3) correspondingly. For the preparation of NPs of core-shell type with different topology, metal ions were reduced sequentially using the molar ratio Ag:Au = 1:1 and were indicated as Au_core_Ag_shell_ and Ag_core_Au_shell_, For all colloids, the initial solutions of tryptophan were adjusted to pH = 10. Thus, amino acid was used in an anionic state that exists in alkaline medium. The pH control of the medium was performed using a pH meter I-160MI. As a working electrode, a glass electrode was used. Silver chloride electrode served as a reference one. Metals were injected into boiling solution of Trp followed by continuous heating of the reaction mixture.

The absorption spectra of the colloidal solutions of AgAu NPs were recorded in the UV-visible region with a spectrophotometer Lambda 35 (Perkin-Elmer, USA) in 1-cm quartz cells.

The size and morphology of nanoparticles were characterized with a transmission electron microscope (TEM) JEM-1230 (JEOL, Japan) with an accelerating voltage of 80 kV. One microliter of colloid was placed on carbon-coated copper grids and dried at room temperature. For the calculation of particle size distribution, the program ImageJ was used.

Scanning electron microscopy (SEM) was performed using a Tescan Mira 3 LMU microscope with an accelerating voltage of 5–20 kV. Energy-dispersive X-ray (EDX) spectra were taken using a built-in Oxford X-max 80 mm^2^ setup.

The particle size distribution function was studied by a laser correlation spectrometer Zetasizer Nano S (Malvern, UK) equipped with a correlator (multicomputing correlator type 7032 ce) by a method based on the scattering of light on any micro-objects. The information signal from the random movement of nanoparticles was analyzed by multichannel spectrum analyzer and colorimeters; 1 ml of studied suspension was placed in a cylindrical optical glass cell with a diameter of 10 mm, which was located in a thermostated sample holder of a laser correlation spectrometer. Registration and statistical processing of the scattered laser light at 173° from the suspension (helium-neon laser LGN-111 was used with power output of 25 mW and wavelength of 633 nm) was performed three times during 120 s at 25 °C. The resulting autocorrelation function was treated with standard computer programs PCS–Size mode v 1.61.

### Animal Husbandry and NP Administration

All the mice employed in our studies were congenic in the C57BL/6J genetic background. Male mice aged 12 weeks and weighing 18–20 g were used in the experiments. The animals were housed in cages containing a sterile paddy husk as bedding in a ventilated animal facility with a controlled temperature (21 ± 2 °C). All the mice were maintained on a laboratory complete diet with free access to water. As a model of malignant tumor growth, Lewis lung carcinoma (LLC) was employed. Tumor strain was kindly provided by the National Bank of Cell Lines from Human and Animal Tissues (R. E. Kavetsky Institute of Experimental Pathology, Oncology and Radiobiology, NAS of Ukraine). LLC transplantation was performed by intra-muscular injection into a femoral muscle of 0.2 ml 10% cell suspension in saline (3 × 10^6^ cells/ml). Starting from day 5 after tumor cell inoculation (the moment of development of palpable tumors), the animals were randomly divided into groups receiving daily an intra-peritoneal injection of 100 μL of the colloidal solution of NPs (either bimetallic alloy NPs with different metal molar ratios, where Ag:Au = 1:1, 1:3, and 3:1, and core-shell NPs with different topology Au_core_Ag_shell_, Ag_core_Au_shell_, where Ag:Au = 1:1) at a dose of 500 μg/kg/day for 12 days. Control group of mice received an intra-peritoneal injection of 100 μL of saline. General morphological parameters (animal weight, primary tumor size) and survival rate were monitored daily.

Twenty-four hours after the last NP injection (day 18 after tumor cells inoculation), the mice underwent euthanasia. At the time of sacrifice, mice were weighed, blood was taken from the inferior vena cava, and liver, lungs, and primary tumor were immediately removed. Blood serum was collected after centrifugation at 1500×*g* for 10 min. To isolate primary peripheral blood lymphocytes, the blood was first layered onto Ficoll-Paque (1.0777 g/ml; Pharmacia) and sedimented at 1000×*g* for 20 min at 21 °C. Mononuclear cells were harvested from the interface and pelleted three times in phosphate-buffered saline (PBS) without Ca^2+^ and Mg^2+^ to remove platelets (first at 650×*g* then twice at 235×*g*).

### NP Antitumor Activity Analysis

Antitumor and antimetastatic activities of the NPs were evaluated on day 18 after tumor transplantation based on morphological parameters of primary tumor volume and number and size of lung metastases [15]. Primary tumor diameter was measured three times per week starting from day 5 after tumor inoculation. Tumor volume (*V*) was calculated by the formula *V* = 0.52*d* ^3^, where *d* is the tumor diameter [15]. Anticancer activity was evaluated as tumor growth inhibition (TGI) rate calculated by the formula

$$ \mathrm{T}\mathrm{G}\mathrm{I}=\frac{V_0-{V}_1}{V_0}\cdot 100\%, $$

where *V*_0_ and *V*_1_ are average tumor volumes in animals of a control and experimental group respectively.

The number and size of lung metastases were routinely analyzed using binocular microscope and millimeter scale. Lung metastasis inhibition (MI) index was calculated by a formula

$$ \mathrm{M}\mathrm{I}=\frac{A_0\cdot {B}_0-{A}_1\cdot {B}_1}{A_0\cdot {B}_0}\cdot 100\%, $$

where *A*_0_ and *A*_1_ are metastasis frequencies in a control and experimental group and *B*_0_ and *B*_1_ are the average number of metastases in lungs of animals in a control and experimental group, respectively.

Subsequently, lung metastasis growth inhibition (MGI) index was calculated by a formula

$$ \mathrm{M}\mathrm{G}\mathrm{I}=\frac{S_0-{S}_1}{S_0}\times 100\%, $$

where *S*_0_ and *S*_1_ are the average metastasis size in a control and experimental group, respectively.

### Serum Lactate Dehydrogenase Activity

As a functional indicator for the monitoring of the activity of tumor growth and tissue infiltration rate, blood serum lactate dehydrogenase (LDH, EC 1.1.1.27) test was used. Lactate dehydrogenase enzymatic activity (IU/l) was determined in blood serum using commercially available kits (Felicit Diagnostics, Ukraine) according to manufacturer’s protocol.

### DNA Comet Assay

A single-cell DNA electrophoresis (DNA comet assay) was employed in order to assess NPs cytotoxicity towards mouse lymphocytes in vivo*.* Comet assay was performed exactly as described in [16]. Comets were visualized using a light microscope at ×400 magnification, and images were captured with a digital camera. Typically, 100 sequentially observed cells were analyzed per sample. Comet tail moment as a product of the tail length and the fraction of total DNA in the tail was then calculated automatically using *TriTek CometScore* ^*TM*^ software. Results were expressed as a percentage distribution of comets with a certain tail moment.

### Oxidative Damage Product Measurement

The degree of oxidative modification of hepatic and primary tumor tissue proteins was assessed as previously described [14] through assessment of the levels of protein carbonylation and of protein sulfhydryl groups. Lipid peroxidation in hepatic and primary tumor tissue was determined by assessing the level of thiobarbituric acid-reactive substances (TBRAS).

### Statistical Analysis

All data are presented as means ± S.D. Statistical comparisons involving treatment and control groups were first analyzed by a one-way ANOVA followed by multiple comparisons employing Tukey’s HSD post hoc test. *P* values less than 0.05 were considered to be statistically significant.

## Results and Discussion

### Bimetallic Nanoparticles

Bimetallic AgAu nanoparticles were synthesized in the presence of the essential amino acid tryptophan as a reducing/stabilizing agent aiming to reduce toxicity of nanosized metals compared to those obtained in the presence of strong reducing agents and surfactants, as was previously demonstrated [14]. Tryptophan in an aqueous solution adjusted to high pH exists in anionic form having nonbonding pair of electrons on the nitrogen atom of the amino group and deprotonated carboxylic group -COO^−^. These groups are involved in donor-acceptor bond formation with *d*-orbitals of the metal. During the synthesis of NPs, two competing processes occur, donor-acceptor complex formation and metal reduction through indole ring. Thus, one molecule of Trp can serve as a bridge between NPs causing their aggregation. Reduction of the noble metal with tryptophan leads to the formation of large aggregates of NPs. To avoid such process, an excess of amino acid was used.

**Fig. 1:**
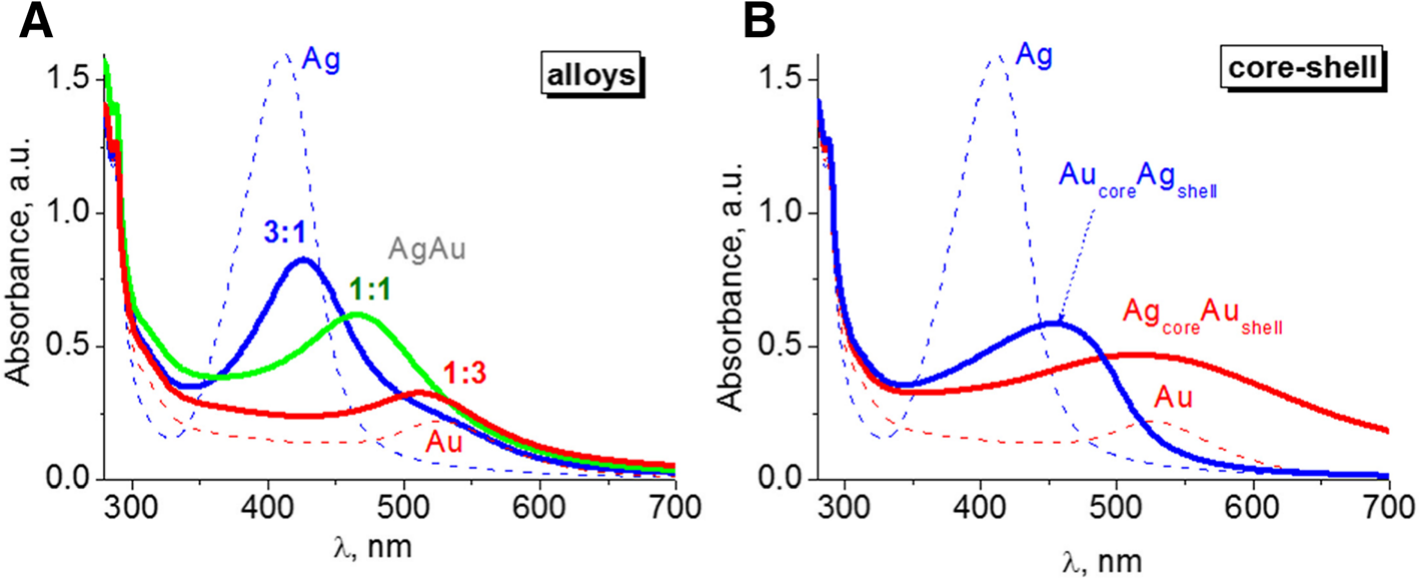
Optical spectra of colloidal solutions of bimetallic NPs of alloy type with different metal molar ratios (**a**) and core-shell type with different topology (**b**)

Colloidal solutions of obtained NPs had a bright color due to the localized surface plasmon resonance (LSPR) band absorption. Only one LSPR band is characteristic for bimetallic alloy NPs formed when Ag^+^ and [AuCl_4_]^−^ ions are simultaneously reduced. Its maximum is located between the maxima inherent to the LSPR bands of individual metals (Fig. 1a) and is shifted from silver to gold with decreasing Ag:Au molar ratio. Namely, band maxima of AgAu NPs were observed at 427 nm for the ratio of 3:1, 466 nm for 1:1, and 511 nm for 1:3, between LSPR bands of individual metals obtained in the same experimental conditions at 411 nm for nanosized silver and 526 nm for gold.

**Fig. 2:**
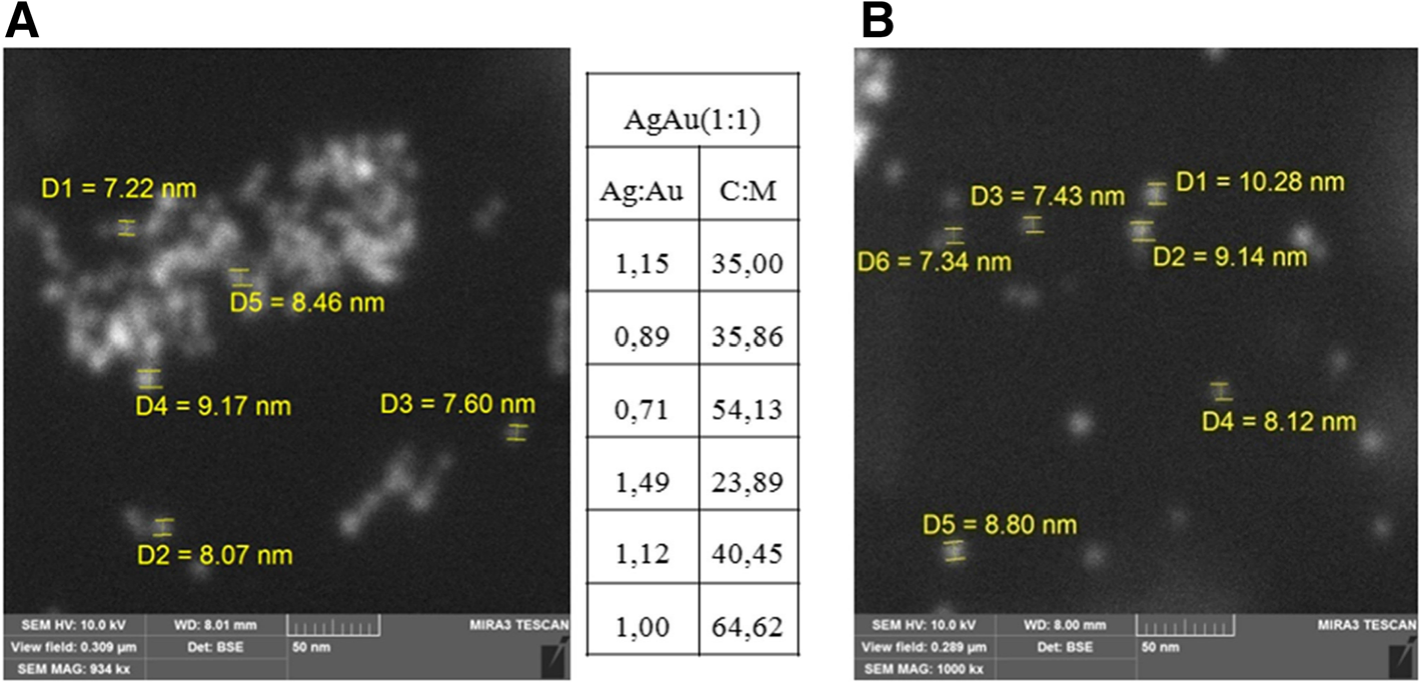
SEM images of alloy NPs: sample AgAu(1:1) (**a**), *inset* shows atomic ratio of metals Ag:Au and carbon to metal ratio according to SEM/EDX measurements; sample AgAu(1:3) (**b**), scale 50 nm

According to SEM images, the average size of AgAu NPs of alloy type was of 7–10 nm (Fig. 2a). We used elemental analysis to check the content of metals in obtained NPs. For the sample AgAu(1:1) studied with SEM/EDX, the Ag:Au ratio corresponds to a given experimental quantity. Namely, the average value shown in atomic % ratio was Ag:Au_average_ = 1 (Fig. 2a, inset). Also, we fixed amount of carbon that was added to the mixture in the form of tryptophan. The experimental molar ratio Trp:M = 2:1 corresponds to atomic ratio C:M = 22:1, where C—carbon and M—metal. SEM/EDX showed that in the area of nanoparticles, carbon quantity exceeds a given number by several times, suggesting the accumulation of amino acid and its fragments around NPs, and thus forming an organic stabilizing shell.

**Fig. 3:**
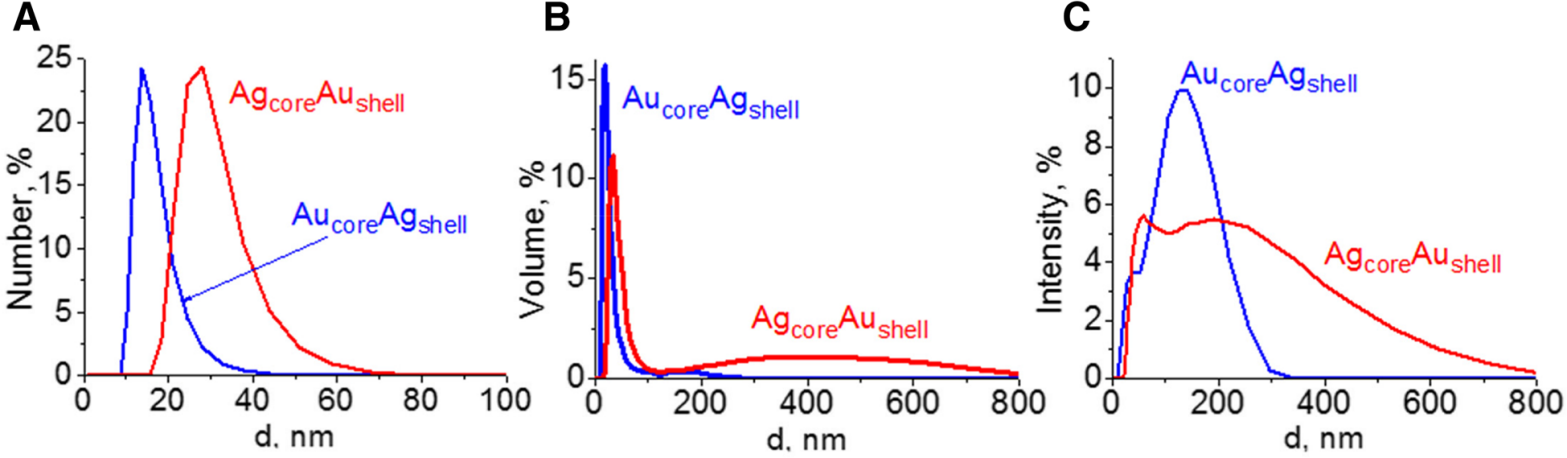
The nanoparticle size distribution of Au_core_Ag_shell_ and Ag_core_Au_shell_ NPs by number (**a**), volume (**b**), and intensity (**c**) basis obtained with DLS method

Bimetallic core-shell NPs were synthesized by sequential reduction of metal ions. The disappearance of LSPR absorption band of previously formed monometallic particles, used as a core, and the growth of LSPR band of the second metal in the spectra of the same colloid indicates the occurrence of new plasmonic metal shell. Such process is what we observed for both systems—previously formed silver core with a new gold shell and vice versa (Fig. 1b). High-intensive characteristic band of Ag NPs at 411 nm is screened by the absorption of gold nanoparticles at 515 nm. Also, LSPR band of Au NPs at 526 nm is covered by silver absorption with a maximum at 454 nm. Broad LSPR bands as well as their asymmetry indicate polydispersity and association of NPs of core-shell type in colloidal solutions. We suggest that it can be the aggregation of small NPs occurring due to cross-linking of particles through tryptophan during the synthesis. Polydispersity in colloids was evidenced by the dynamic light scattering (DLS) data (Fig. 3). Volume and intensity basis suggested the formation of a wide range of structures up to 300 nm in case of Au_core_Ag_shell_. And large aggregates up to 800 nm are characteristic for Ag_core_Au_shell_. At the same time, there are big fractions of small particles with an average diameter of 15 and 30 nm correspondingly.

**Fig. 4:**
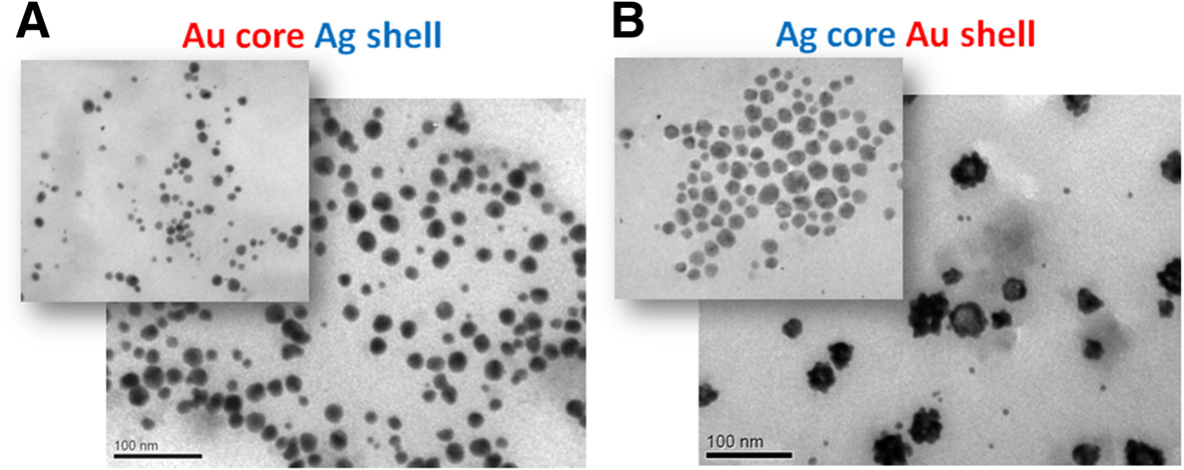
The TEM images of bimetallic NPs of core-shell type: Au_core_Ag_shell_ (**a**) and Ag_core_Au_shell_ (**b**). The *insets* show previously formed monometallic NPs applied as a core. Scale 100 nm

The clear difference in topology of core-shell NPs is confirmed by TEM measurements. Au_core_Ag_shell_ NPs are mainly spherical particles of 10–25 nm (Fig. 4) while their gold core (previously formed monometallic Au NPs) was smaller with the average size of 5–10 nm. Thus, as expected, the growth of the shell is accompanied by an increase of particle size. The same trend is characteristic for the system with silver core of 10–25 nm, and twice bigger Ag_core_Au_shell_ NPs of 25–50 nm, but in this case, the shell is not uniform. According to TEM, the silver cores are mainly covered by small gold particles. The presence of small Au NPs of 5–10 nm in colloid presumably confirms that the formation of gold particles in the presence of tryptophan occurs simultaneously with the gold shell growth around Ag core due to fast reduction of gold with amino acid. Moreover, it causes an aggregation shown by TEM as well as by DLS.

### Antitumor Activity

An in vivo antitumor activity of the administered NPs was estimated based on the rate of primary tumor growth inhibition (TGI), lung metastasis growth inhibition (MGI), and metastasis inhibition (MI) indexes [15]. An inhibitory effect of NPs towards LLC primary tumor growth was observed for mice treated with NPs either with different Ag:Au molar ratio (AgAu(1:3), AgAu(1:1), AgAu(3:1)) or with different topology (Au_core_Ag_shell_, Ag_core_Au_shell_) (Table 1). Based on a primary tumor growth inhibition rate, all the administered NPs possessed an antitumor activity in vivo. For the majority of AgAu NPs administered, primary tumor growth inhibition was more than 30%. The maximum value (34%) of LLC primary tumor inhibition was attributed to NP with metal molar ratio as 1:3, AgAu(1:3) (Table 1).

**Table 1:** The indexes of antitumor activity of gold/silver bimetallic nanoparticles

Group	Type of nanoparticles	Primary tumor growth inhibition, %	Metastasis inhibition index, %	Lung metastasis growth inhibition, %
I	AgAu(1:3)	34	−28	4
II	AgAu(1:1)	33	−10	1
III	AgAu(3:1)	26	19	−36
IV	Au_core_Ag_shell_	32	−22	8
V	Ag_core_Au_shell_	31	13	27

Despite a primary tumor growth inhibition, the administered NPs were not efficient in inhibiting of LLC lung metastases development. Metastasis inhibition (MI) index was calculated based on a frequency and a number of metastatic nodes in mouse lungs. During the administration of AgAu(3:1) and Ag_core_Au_shell_ NPs, MI index was 19 and 13%, respectively, whereas the treatment of mice with AgAu(1:3) (−28%), Au_core_Ag_shell_ (−22%), and AgAu(1:1) (−10%) was characterized even by stimulation of metastasizing (Table 1).

Lung metastases were observed in mice of all the treatment groups. Lung metastasis growth inhibition index ranged from 4% (in AgAu(1:3)-treated group) to 27% (in Ag_core_Au_shell_-treated group). Conversely, the treatment with AgAu(3:1) NPs led to an accelerated metastasis growth in mice (MGI = −36%).

Thus, based on the parameters of primary tumor growth inhibition, lung metastasis growth inhibition, and metastasis inhibition index, it can be concluded that the highest antitumor activity was attributed to Ag_core_Au_shell_ NPs (Table 1).

**Fig. 5:**
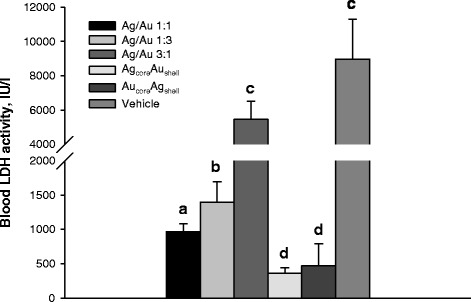
Blood serum lactate dehydrogenase (*LDH*) activity in mice with LLC following NP administration. LDH activity was assessed in serum of mice on day 18 after tumor cell inoculation and after daily intra-peritoneal injections of NPs for 12 days at a dose 500 μg/kg/day. Values marked with *different letters* (*a*, *b*, *c*, *d*) are statistically different, *P* < 0.05. All values are given as the mean ± 1 S.D., *n* = 5 for each group

NPs administration to mice was accompanied with low serum lactate dehydrogenase (LDH) activity, an indicator of tumor growth activity and antitumor treatment effectiveness [17]. For the mice with LLC that were not treated with NP, the level of serum LDH activity was more than 8500 IU/I (Fig. 5). As seen from the Fig. 5, NP administration led to a reduction of serum LDH activity supporting an observed inhibitory effect of NP administration towards tumor growth. The lowest value of LDH activity was detected in serum of mice treated with Ag_core_Au_shell_ and Au_core_Ag_shell_. At the same time in the groups characterized with comparably higher primary tumor growth inhibition rates, serum LDH activity tended to be significantly lower. However, AgAu(3:1) NP administration had no significant effect on serum LDH activity in mice with LLC. This was consistent given the lowest primary tumor growth inhibition rate in this treatment group (Table 1, Fig. 5).

Oxidative stress within the cells is considered as one of the primary mechanisms underlying NP antitumor activity [18–20]. Silver and gold nanoparticles were reported to have an intrinsic property that allows a Fenton-type reaction to produce ROS [21, 22] with a subsequent lipid and protein oxidation, DNA damage, and cell death induction [23]. Several studies underscored a direct interaction between the ability of NPs to initiate ROS production and the subsequent cell death [2, 23]. Our results are consistent with this paradigm showing that administration of NPs resulted in oxidative stress induction in primary tumor tissue as was evidenced by accumulation of protein oxidation markers. However, based on the data, it can be concluded that only protein carbonylation may have an impact on primary tumor growth rates and metastasizing.

**Fig. 6:**
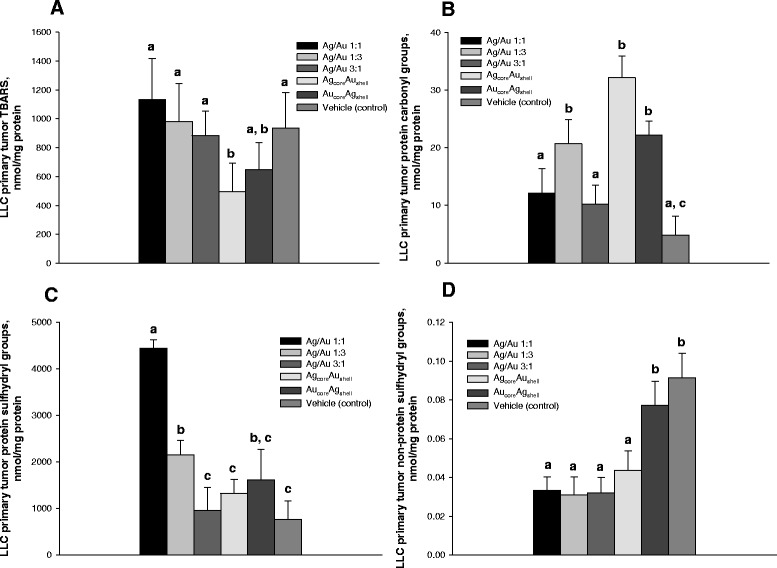
Oxidative damage products in LLC primary tumor tissue following NP administration to mice. Levels of thiobarbituric acid reactive substances (**A**), protein carbonyl (**B**), and thiol (**C**) groups and nonprotein thiol groups (**D**) were determined in primary tumor tissue homogenates on day 18 after tumor cell inoculation and after daily intra-peritoneal injections of NPs for 12 days at a dose 500 μg/kg/day. Values marked with *different letters* (*a*, *b*, *c*) are statistically different, *P* < 0.05. All values are given as the mean ± 1 S.D., *n* = 5 for each group

The analysis of oxidative processes intensity in the primary tumor tissue was performed by determining lipid and protein oxidative damage product accumulation. Consistent with morphological parameters of antitumor activity, we observed higher levels of oxidative damage product accumulation in primary tumor tissue of mice treated with NPs (Fig. 6). NP treatment did not accelerate lipid oxidation in the primary tumor (Fig. 6A), whereas led to higher protein oxidation (Fig. 6B). The administration of NPs with well-defined topology of metal components (either Ag_core_Au_shell_ or Au_core_Ag_shell_) was the most sufficient in inducing protein carbonylation in LLC primary tumor tissue. Conversely, the use of NPs with different metal molar ratios (AgAu(1:1) and AgAu(3:1)) did not result in higher tumor protein carbonylation and did not affect or even cause elevation of primary tumor protein sulfhydryl groups (Fig. 6C). Unexpectedly, the action of NPs in case of AgAu(1:1), AgAu(1:3), and Au_core_Ag_shell_ was accompanied with even significantly higher levels of protein thiol groups. However, the administration of NPs, except for the Au_core_Ag_shell_ NPs, resulted in significantly lower levels of nonprotein thiol groups in primary tumor tissue (Fig. 6D).

One of the major limitations of any substances with antitumor activity is a side effect expressed in cytotoxicity towards untransformed cells and tissues [20]. Since both therapeutic and toxic effects of nanoparticles need to be studied simultaneously, we have analyzed the ability of NPs to induce hepatic lipid and protein oxidation as well as lymphocyte DNA stability.

**Fig. 7:**
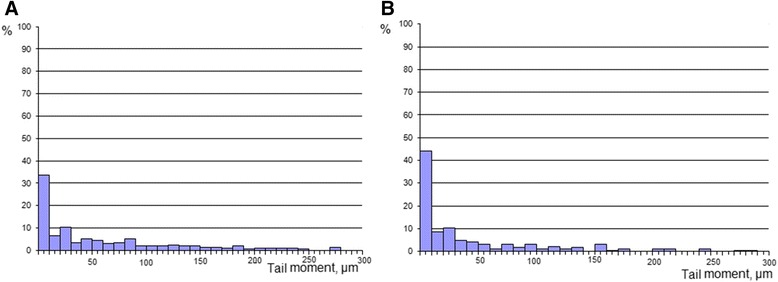
Tail moment distribution of primary lymphocyte DNA comets of mice with LLC following bimetallic NP administration. DNA comet assay was performed employing primary lymphocytes isolated from mice with LLC following AgAu(3:1) (**a**) and Ag_core_Au_shell_ (**b**) bimetallic NP administration; *X*-axis—the length of tail moment, μm; *Y*-axis—percentage of the comets

Our attention was predominantly focused on Ag_core_Au_shell_ NPs as these showed the highest antitumor activity. Ag_core_Au_shell_ NPs upon their administration to mice with LLC possessed low in vivo genotoxicity towards untransformed cells as evidenced by single-cell DNA comet analysis of primary mouse lymphocytes (Fig. 7). The analysis of comet moment distribution revealed that up to 70% of cell population was characterized with intact integrated nuclear DNA after NPs administration. The percentage of cells with a damaged DNA (tail moment more than 150 μm) was only 10% (Fig. 7). Conversely, in a group of mice treated with AgAu(3:1) NPs, only 59% of the cells had intact nuclear DNA; 25% of the comets analyzed in this group had tail moment more than 150 μm, suggesting higher genotoxicity of these NPs towards lymphocytes.

**Fig. 8:**
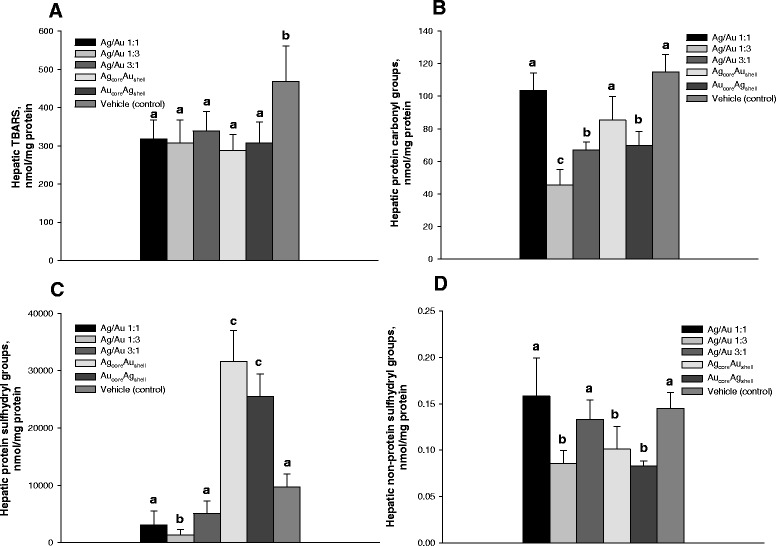
Oxidative damage products in livers following NP administration to mice. Levels of thiobarbituric acid reactive substances (**A**), protein carbonyl (**B**), and thiol (**C**) groups and nonprotein thiol groups (**D**) were determined in liver homogenates on day 18 after tumor cell inoculation and after daily intra-peritoneal injections of NPs for 12 days at a dose 500 μg/kg/day. Values marked with *different letters* (*a*, *b*, *c*, *d*) are statistically different, *P* < 0.05. All values are given as the mean ± 1 S.D., *n* = 5 for each group

All of the NPs administered in the present study did not lead to an increased hepatic lipid oxidation (Fig. 8A). After Ag_core_Au_shell_ and Au_core_Ag_shell_ NP treatment, the livers retained even higher protein sulfhydryl group levels (Fig. 8C), lower level of protein carbonylation (Fig. 8B) compared to the same parameters in the livers of untreated mice with LLC. These data suggest lower levels of oxidative damage in the livers of mice with LLC receiving NPs.

The data generated in the current study allow for concluding the presence of antitumor activity of bimetallic gold and silver NPs. Among the tested samples, and especially among the NPs with the same metal ratio of Ag:Au = 1:1 (alloy and core-shell type), NPs that consisted of Ag core covered by Au shell were the most active towards LLC tumor growth and metastasizing inhibition and possessed the lowest toxicity. Thus, the studied biological activity was the highest in case of ordered topological distribution of metal compounds within Ag_core_Au_shell_ NPs, suggesting that in vivo antitumor activity of the studied NPs is strongly dependent on gold and silver interaction arising from their ordered topological distribution.

## Conclusions

Based on the parameters of primary tumor growth inhibition, lung metastasis growth inhibition, and metastasis inhibition index, the highest antitumor activity was attributed to Ag_core_Au_shell_ NPs among the studied series of bimetallic nanoparticles with different metal content and topology. This was also confirmed by low serum lactate dehydrogenase activity compared to untreated mice with LLC and higher tumor tissue protein carbonylation. Ag_core_Au_shell_ NPs showed low genotoxicity towards mouse lymphocytes as evidenced by DNA comet analysis and did not affect hepatic lipid and protein oxidation. Our data suggest that Ag_core_Au_shell_ NPs used in this study may serve as a suitable prototype to develop anticarcinomatous agent and anticancer drug vehicle.

## Abbreviations

DLS: Dynamic light scattering
LDH: Lactate dehydrogenase
LLC: Lewis lung carcinoma
LSPR: Localized surface plasmon resonance
MGI: Metastasis growth inhibition
MI: Metastasis inhibition
NPs: Nanoparticles
ROS: Reactive oxygen species
TGI: Tumor growth inhibition
Trp: Tryptophan
